# Sessile serrated lesions with dysplasia: is it possible to nip them in the bud?

**DOI:** 10.1007/s00535-023-02003-9

**Published:** 2023-05-23

**Authors:** Takahiro Utsumi, Yosuke Yamada, Maria Teresa Diaz-Meco, Jorge Moscat, Yuki Nakanishi

**Affiliations:** 1 Department of Gastroenterology and Hepatology, Kyoto University Graduate School of Medicine, 54 Kawaharacho, Shogoin, Sakyo-Ku, Kyoto, 606-8507 Japan; 2 Department of Diagnostic Pathology, Kyoto University Hospital, Kyoto, Japan; 3 Department of Pathology and Laboratory Medicine, Weill Cornell Medicine, New York City, NY USA

**Keywords:** Serrated pathway, SSL, SSLD, Classification, Tumor microenvironment

## Abstract

The serrated neoplasia pathway constitutes an “alternative route” to colorectal cancer (CRC), and sessile serrated lesions with dysplasia (SSLDs) are an intermediate step between sessile serrated lesions (SSLs) and invasive CRC in this pathway. While SSLs show indolent growth before becoming dysplastic (> 10–15 years), SSLDs are considered to rapidly progress to either immunogenic microsatellite instable-high (MSI-H) CRC (presumably 75% of cases) or mesenchymal microsatellite stable (MSS) CRC. Their flat shapes and the relatively short window of this intermediate state make it difficult to detect and diagnose SSLDs; thus, these lesions are potent precursors of post-colonoscopy/interval cancers. Confusing terminology and the lack of longitudinal observation data of serrated polyps have hampered the accumulation of knowledge about SSLDs; however, a growing body of evidence has started to clarify their characteristics and biology. Together with recent efforts to incorporate terminology, histological studies of SSLDs have identified distinct dysplastic patterns and revealed alterations in the tumor microenvironment (TME). Molecular studies at the single-cell level have identified distinct gene alterations in both the epithelium and the TME. Mouse serrated tumor models have demonstrated the importance of TME in disease progression. Advances in colonoscopy provide clues to distinguish pre-malignant from non-malignant-SSLs. Recent progress in all aspects of the field has enhanced our understanding of the biology of SSLDs. The aim of this review article was to assess the current knowledge of SSLDs and highlight their clinical implications.

## Introduction

Colorectal cancer (CRC) is the third most common malignancy worldwide and the second leading cause of cancer-related death [1]. Colonoscopy is used to detect and simultaneously remove pre-malignant colorectal polyps before they develop into invasive cancers. However, several studies have reported that 5–8% of all CRCs are diagnosed in patients who have undergone colonoscopies 3–5 years before diagnosis [2–6]. These cancers are usually called post-colonoscopy CRCs or interval CRCs, and have become an important clinical issue that attracts physicians’ attention.

Three decades ago, sporadic CRCs were considered to arise exclusively from colorectal adenomas through the “adenoma–carcinoma sequence,” and colorectal serrated polyps, characterized by a saw-toothed pattern of colonic crypts, were thought to be harmless hyperplastic lesions [7, 8]. However, serrated polyps have now been recognized as alternative precursors that potentially progress to CRC, representing another key oncogenic route, named “serrated neoplasia pathway,” which accounts for approximately 15–30% of sporadic CRCs [9–13]. In this alternative route, CRCs mostly arise from sessile serrated lesions (SSLs), which display a flat elevated hyperplastic mucosa with a unique morphological change in crypt bottom, such as a “boot” or “inverted T” shape appearance. After gradual growth for a long period (typically over 10–15 years), SSLs begin to contain malignant dysplastic areas (SSLs with dysplasia: SSLDs) that presumably progress to CRCs within a short duration [14, 15]. Thus, SSLDs are an intermediate state between SSLs and CRCs and exist for a very short period during the serrated pathway. Their flat shapes make it difficult to detect SSLs and SSLDs by colonoscopy or other modalities such as computed tomography (CT) or CT colonography and causes incomplete endoscopic resection for their removal [16–20]. Therefore, SSLs/SSLDs are considered a threat precursor of post-colonoscopy/interval cancers that share many genomic and colonic site characteristics with SSLDs [2, 17]. However, the bona fide malignant potential of SSLs (i.e., the proportion of SSLs that truly progress to advanced CRCs via SSLDs) has not been revealed because of the lack of reliable longitudinal observational data on the natural history of SSLs. In addition, the clinicopathological and molecular features of SSLDs are not well defined. Furthermore, despite recent advancements in understanding the role of the tumor microenvironment (TME) in CRC development and its therapeutic application [21–23], alterations in the TME of SSLs/SSLDs remain to be elucidated. Therefore, a full clarification of the distinct characteristics of SSLDs on molecular, histological, and endoscopic bases is vital to develop a method to distinguish SSLs that are directed toward invasive CRC.

In this review, after a brief introduction of each subtype of serrated polyps, we describe the current knowledge of epidemiology, histological, and endoscopic characteristics, and optimal therapeutic indications of SSLDs, with a particular focus on the difference between non-malignant SSLs and SSLDs. In addition, molecular and microenvironmental alterations, which are proposed to contribute to the progression from SSL to dysplasia and cancer, are discussed, highlighting new findings from the recent literature.

## Classification of serrated polyps

The latest World Health Organization (WHO) classification released in 2019 divides serrated polyps histopathologically into “hyperplastic polyps (**HPs**)”, “sessile serrated lesions (**SSLs**)”, “traditional serrated adenomas (**TSAs**)”, and “serrated adenoma, unclassified” [24] (Table 1).

**Table 1:** The classification of serrated colorectal lesions according to World Health Organization classification 5th edition (2019)

The classification of serrated colorectal lesions according to 2019 WHO classification	
Histological type	Subtype
Hyperplastic polyp (HP)	Microvescicular type (MVHP)
	Goblet cell-rich type (GCHP)
Sessile serrated lesion (SSL) SSL with dysplasia (SSLD)	
Traditional serrated adenoma (TSA)	
Serrated adenoma, unclassified	

*WHO* World Health Organization

HPs are the most common, accounting for approximately 75% of all serrated polyps, and are generally considered benign [25]. HPs are usually found in distal colons (left-sided) of ≤ 5 mm in size and are characterized by the elongation of the intestinal crypts, with serration of the upper part of the crypts and uniform proliferation of the basal part of the crypts. Additionally, HPs have two variants: microvesicular hyperplastic polyps (MVHPs) and goblet cell-rich hyperplastic polyps (GCHPs) [24]. MVHPs are characterized by small droplets of mucin, whereas GCHPs are characterized by an increased number of goblet cells. MVHPs are considered to progress to SSLs [10, 26].

Both SSLs and TSAs are now recognized as precursors of CRCs, but SSLs are expected to be a more significant contributor to the burden of CRCs [15]. SSLs are generally larger than HPs and are more frequently detected in the proximal colon (right-sided) [27, 28]. In addition, SSLs have distinctive molecular features, including hypermethylation of CpG islands in the promoter regions of tumor suppressor genes, and *BRAF* mutations [29, 30]. SSLs with dysplasia (SSLDs) represent an intermediate step between SSLs and advanced CRCs and are considered to progress to either of the following subtypes: microsatellite instable-high (MSI-H) CRC with *MLH1* inactivation or microsatellite stable (MSS) CRC without *MLH1* alteration [25, 31–33].

TSAs are the least frequent type of serrated polyps, accounting for < 1% of all polyps, and are commonly located in the distal colon (left-sided), similar to the localization of HPs [34–36]. TSAs often demonstrate villous architecture with cells that contain prominent eosinophilic cytoplasm and penicillate nuclei, and are endoscopically characterized by a “pinecone-like” or “branch coral-like” appearance [37, 38]. TSAs can progress to MSS CRCs with distinct molecular features such as CIMP-high and *BRAF* mutations without *MLH1* inactivation or *KRAS* mutations without any CIMP-high, *BRAF* mutations, or *MLH1* inactivation [38–40].

“Serrated adenoma, unclassified” has been introduced to be used for rare ambiguous colorectal polyps showing both adenomatous and serrated architecture, which cannot be clearly classified as SSL, TSA, or conventional adenoma [24]. Recently, superficially serrated adenoma (SuSA), characterized by mixed adenomatous and serrated features with superficially spread, has been reportedly related to a subtype of TSA [41–43].

Hereafter, we will focus on the neoplasia pathway occurring from SSL through SSLD, which is the most common precursor of serrated CRC.

## Epidemiology of SSLs and SSLDs

SSLs account for up to 8% of colorectal polyps in the screening population, and approximately 25% of serrated polyps [25, 34, 44–46]. Most SSLs are < 1 cm in size, frequently located in the proximal colon, and have a female predominance [25, 37]. However, the true prevalence of SSLs remains unknown because of the difficulty in detection due to their obscure appearance and poor discrimination from other types of polyps by colonoscopy. In addition, resected specimens may not be adequately diagnosed as SSLs owing to inconsistent diagnostic criteria and terminology for serrated lesions among pathologists [9, 11, 47].

**Fig. 1:**
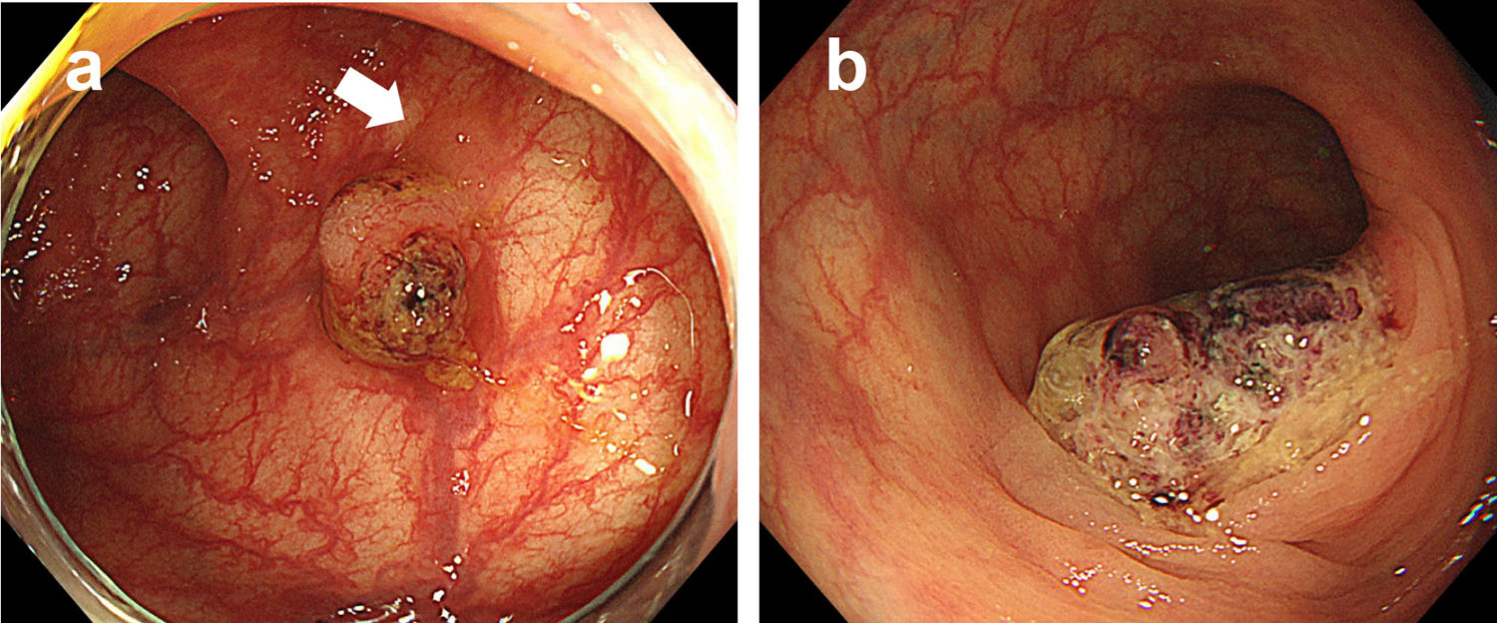
Case of suspected rapid progression from a sessile serrated lesion with dysplasia (SSLD) to advanced cancer. **a** A 5 mm sized sessile-type lesion with stool and mucus adhesion, located in the transverse colon. A 3 mm sized area of no vessel visibility (white arrow) accompanying the lesion suggests the presence of a flat serrated lesion. The lesion was retrospectively diagnosed as an SSLD of 8 mm in diameter. **b** The patient had anticoagulant therapy and did not give consent for immediate endoscopic treatment. After 8 months, follow-up endoscopy showed 20 mm sized Type 1 (polypoid type) advanced colon cancer with a loss of surface glandular structure. Postoperative diagnosis was advanced colon cancer (pT2N1M0 and pStage IIIA, UICC)

SSLDs are not frequently found, occurring in approximately 0.5% of average-risk patients (approximately 4–8% of all SSLs) [25, 44, 45, 48]. However, this observation does not necessarily indicate that SSLs rarely develop into CRCs; rather, it may reflect a relatively short window of this intermediate state for detection compared with that of SSLs or conventional adenomas. Once dysplasia occurs in SSLs, these lesions may have the potential to rapidly transform into invasive CRCs. Several reports have demonstrated cases of rapid progression from SSLDs to advanced cancers in a short period of time [49–51] (Fig. 1). Bettington et al. reported that the dwell time of SSLs before dysplasia occurred was approximately 17 years, whereas there was no significant difference in age between patients with SSLDs and those with carcinoma, supporting the theory that this intermediate state lasts only for a short time before full transformation of the lesions [15]. Difficulties in both endoscopic detection and resection and pathological evaluation may lead to misdiagnosis of SSLDs. For example, the endoscopic miss rate is high for SSLDs, whereas the complete excised lesion rate is low [17, 19, 47]. Pathologists find it difficult to distinguish SSLs with extensive dysplasia from conventional adenomas. Furthermore, since dysplastic areas within SSLDs are often polypoid while the non-dysplastic part in the same lesion is flat, there is a potential issue that endoscopists may resect only the polypoid portion while leaving the remaining part (which is histologically serrated), which might lead to a wrong diagnosis of a conventional adenoma on pathological examination [17].

SSLDs and SSLs with carcinoma appear to share a site preference (proximal colon) and gender distribution (female predominance) with SSLs without dysplasia [15, 52]. Recent evidence has shown that 80% of 741 SSLs without dysplasia were located in the proximal colon; 55% of SSLs occur in women [27]. Among 137 cases of SSLDs or SSLs with carcinoma, 87% were proximal and 61% occurred in women. The mean polyp size of these SSLDs or SSLs with carcinoma was 10.7 mm, slightly larger than that of SSLs without dysplasia (8.5 mm) [15, 27]. SSLDs are either mismatch repair gene-deficient (MMRD) or mismatch repair gene-proficient (MMRP); approximately 75% of SSLDs are MMRD [53]. Regarding sex distribution and location, a report demonstrated a sharp difference between MMRD and MMRP SSLDs; 70.4% of MMRD SSLDs occurred in women, whereas 36.4% of MMRP SSLDs occurred in women. Only 8.5% of MMRD SSLDs arise in the distal colon or rectum, while this accounts for 28.1% of MMRP SSLDs [15].

## Histological features of SSLs and SSLDs

According to the 2019 WHO classification (5th edition) [24], the characteristic histological features of SSLs include horizontal growth along the muscularis mucosae, dilation of the crypt base (basal third of the crypt), serrations extending into the crypt base, and asymmetrical proliferation. If there is at least one clearly distorted crypt, the lesion is diagnosed as an SSL. Of note, this latest WHO classification recommends the use of term “sessile serrated lesion (SSL)” instead of other terms such as “sessile serrated adenoma (SSA),” “sessile serrated polyp (SSP),” or “sessile serrated adenoma/polyp (SSA/P)”. Since there is significant inter-observer variation in identifying, classifying, and even naming serrated lesions among pathologists [9, 11, 47], this change in diagnostic rule should help increase the sensitivity to detect SSLs and improve the consistency in terminology for the lesions. After this new criterion was introduced, there was a 7% increase in the diagnosis of SSLs [27]. SSLs and sessile serrated polyps defined by other terms are strictly different; however, in the present review, we adopted SSL as a term defining all sessile serrated lesions (i.e., SSA, SSP, SSA/P, and SSL) without dysplasia, regardless of the era of the study.

SSLDs represent an abrupt transition from SSLs and are characterized by the existence of crypt architecture with cytological atypia [24]. Dysplasia occurring in SSLs tends to show rapid progression to carcinoma, even if dysplasia is morphologically low grade. Therefore, applying the same dysplasia grading system used for conventional adenomas is not recommended for SSLDs. Approximately 75% of SSLDs demonstrate a loss of MLH1 staining in dysplastic areas, reflecting the hypermethylation of *MLH1* [53]. Although loss of MLH1 staining is a good indicator for identifying the presence of dysplasia, retained MLH1 staining does not exclude dysplasia. In the 2010 WHO classification [54], SSLDs were classified into two main categories, “dysplasia resembling conventional adenomas” and “serrated dysplasia” (Table 2). Dysplasia resembling conventional adenomas was referred as intestinal dysplasia in the 2019 WHO classification. SSLs with intestinal dysplasia morphologically resemble conventional adenomas, but are distinct from mixed lesions composed of both SSLs and adenomas. However, a substantial proportion of SSLs with intestinal dysplasia may involve collisions between SSLs and conventional adenomas. Analysis of *BRAF* mutation status using molecular testing and BRAF-V600E immunohistochemistry [55] demonstrated that among 13 SSLs with intestinal dysplasia displaying a *BRAF* mutation in their non-dysplastic component, 10 were *BRAF* wild type in their dysplastic component. SSL with serrated dysplasia is characterized by atypical nuclei, prominent nucleoli, eosinophilic cytoplasm, and increased mitotic activity [24].

**Table 2:** The classification of sessile serrated lesions with dysplasia

The classification of SSLDs	
The WHO classification 2010	The new classification proposed by Liu
Dysplasia resembling conventional adenomas	Adenomatous dysplasia Serrated dysplasia
Serrated dysplasia	Minimal deviation dysplasia Dysplasia not otherwise specified

*SSLDs* sessile serrated lesions with dysplasia, *WHO* World Health Organization

**Fig. 2:**
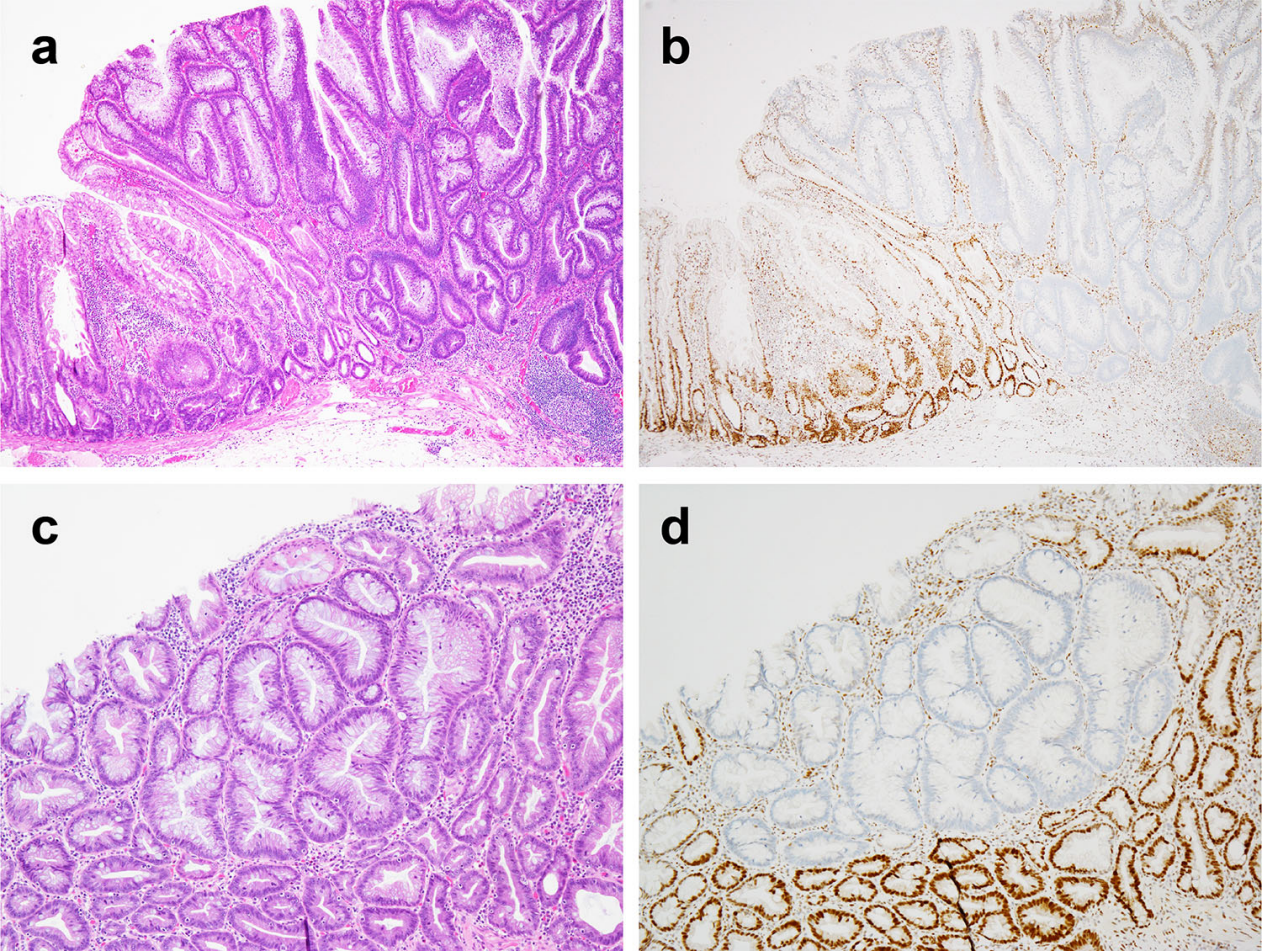
Case of sessile serrated lesion (SSL) with intestinal dysplasia (a, b), accompanied by minimal deviation dysplasia (c, d). **a** Non-dysplastic SSL (left) and intestinal dysplasia resembling the architecture of conventional adenoma (right). **b** Only intestinal dysplasia showed loss of MLH1 staining. (c, d) Minimal deviation dysplasia, showing large glandular structures, hypermucinous change, and loss of MLH1 staining, was incidentally identified apart from the intestinal dysplasia in location

As the 2010 version of the WHO classification was unable to adequately and fully describe the spectrum of morphological dysplasia occurring in SSLs, the updated version (released in 2019) introduced a subtype exhibiting subtle cytological atypia, including hypermucinous changes. Liu et al. identified the presence of other dysplastic patterns occurring in SSLs that do not fall into either intestinal or serrated dysplasia and proposed two more entities, “minimal deviation dysplasia” and “dysplasia not otherwise specified (NOS)” [53] (Table 2). Minimal deviation dysplasia is defined by minor architectural and cytological changes that are often accompanied by the loss of MLH1 expression in SSL (Fig. 2). Although dysplasia has mild disorganization and crowding in the crypt and reduced luminal serration, its architectural and cytological changes are slight; thus, it is difficult to histologically identify the minimal dysplastic area occurring in SSL without MLH1 staining. This subtype accounts for 19% of SSLDs, with a higher incidence than that of intestinal dysplasia (8%) or serrated dysplasia (12%) but is frequently accompanied by other dysplastic patterns. In an analysis of 266 SSLDs using MLH1 staining, 91% of 50 cases with minimal deviation dysplasia had loss of MLH1 expression, and 72% had other patterns of dysplasia [53]. However, most SSLDs show a diverse range of architectural and cytological abnormalities that do not fit into any of the above categories and are defined as “dysplasia not otherwise specified (NOS).” This subtype was also characterized by a high rate of MLH1 loss (83% of 211 cases). Although not recognized as dysplasia in the current classification, the presence of single or small clusters of crypt cells with loss of MLH1 expression within the SSL has been reported [53]. This slight change in the crypt base resembles “cryptal dysplasia” described by Sano et al. [56] and is also accompanied by other types of dysplasia. These small foci with MLH1 loss may be precursors of dysplastic changes in SSLDs; however, their clinicopathological significance remains unclear.

Immune microenvironmental alterations have also been reported in SSLs and SSLDs; however, to date, they have not been comprehensively investigated in contrast to their unique epithelial changes. Rau et al. reported an increase in intra-epithelial lymphocytes (IELs) in SSLDs and found that *MLH1* methylation and IEL counts were independent and robust parameters for the diagnosis of SSLDs [57]. Superficial erosion and acute neutrophil granulocytes may cause reactive changes, potentially leading to dysplasia. Other studies have demonstrated that IEL density increases with the sequential progression from SSLs to CRCs via SSLDs [58]. There was also a correlation between increased IEL density and immune checkpoint (PD-1 and PD-L1) expression with disease progression and MSI status, as MSI-H SSLDs and CRCs had significantly higher IEL density values and PD-1/PD-L1 expression compared with MSS-serrated CRCs, supporting the stepwise dysplasia–carcinoma sequence of serrated carcinogenesis and the hypermutator phenotype of MSI-H lesions. Recent studies using molecular approaches to human lesions and experimental mouse models have identified substantial changes in the TME of SSLDs and SSLs with carcinoma that might contribute to serrated tumorigenesis (see the next section). More in-depth histological evaluation focusing on the TME in serrated lesions is needed to further classify and determine the importance of immune and stromal components in the serrated pathway.

## Molecular signatures of SSL and SSLD

**Fig. 3:**
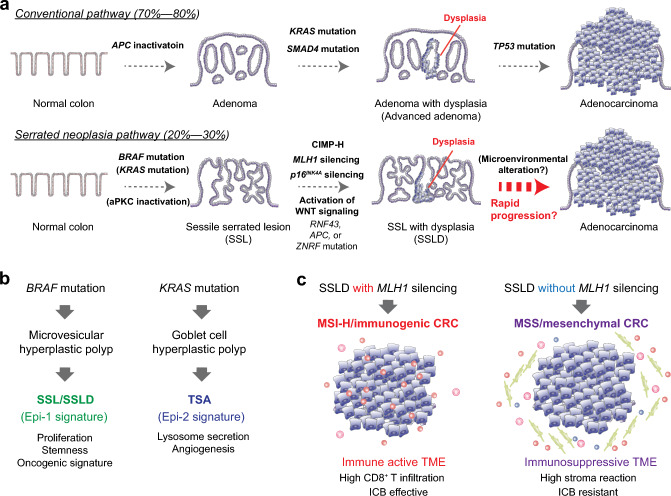
Molecular evolution of sessile serrated lesion with dysplasia (SSLD) and SSLD-derived cancer. **a** Colorectal cancer (CRC) develops through two distinctive pathways. Conventional pathway (top), accounting for 70%–80% of sporadic CRCs, is initiated by inactivation of *APC* in normal cells which results in the formation of conventional-type adenoma. Conventional adenoma acquires the additional mutations of *KRAS*, *SMAD4* and *TP53* which results in the progression to adenoma with dysplasia (advanced adenoma) and finally to adenocarcinoma. Serrated neoplasia pathway (bottom), accounting for 20%–30% of CRCs, is initiated mostly by *BRAF* mutation, which results in the formation of sessile serrated lesion (SSL). Hypermethylation in the CpG island promoter regions (CpG island methylation phenotype; CIMP) of tumor suppressors such as *MLH1* and *p16*^*INK4a*^ results in the silencing of these genes and allow a progression of SSL to SSLD. This process is also associated with the activation of WNT signaling pathway, led by mutation of *RNF43*, *APC* or *ZNRF*. Epigenetic silencing of tumor suppressors and activation of WNT signaling, together with microenvironmental alteration, induces rapid progression of SSLD to adenocarcinoma. **b** *BRAF* mutated lesions develop into SSL/SSLD via microvesicular hyperplastic polyp, while *KRAS* mutated lesions are more likely to develop into traditional serrated adenoma (TSA) via goblet cell hyperplastic polyp. Epi-1 signature is mainly observed in SSL and SSLD, while Epi-2 is exclusively in TSA. Compared to Epi-2, that is characterized by upregulated pathways of lysosome secretion and angiogenesis, Epi-1 demonstrates higher expression of genes related to proliferation, stemness, and oncogenic signatures. **c** Presumable progression of SSLD to CRC, considering their microsatellite status and tumor microenvironment (TME). *BRAF*-mutant SSLD with epigenetic silencing of *MLH1* progress to immunogenic microsatellite instability high (MSI-H) CRC, whereas SSLD without loss of *MLH1* progress to microsatellite stable (MSS) CRC. MSI-H CRC is associated with an immune-active TME and responds better to immune checkpoint blockage (ICB) therapy. MSS CRC of serrated origin is mesenchymal and exhibits high stroma reaction, ICB-resistance, and metastatic behavior with poor prognosis

Analyses of the mutational landscape of serrated lesions have identified an activating *BRAF* mutation as a key alteration in the serrated neoplasia pathway, which is present in 70%–81% of SSLs [59] and results in the constitutive upregulation of the MAPK signaling cascade (Fig. 3a). Mutations in *KRAS* (approximately 9% of SSLs) are also observed in SSLs, but at a much lower frequency than those in *BRAF*. Activation of the MAPK signaling pathway leads to dysregulation of crypt cell proliferation and differentiation, which gives rise to serrated lesions [29, 60–62]. These mutated lesions develop into serrated precursors (microvesicular HPs and SSLs) that are often associated with the hypermethylation of CpG island promoter regions (the CpG island methylation phenotype; CIMP-H), which results in the silencing of a number of tumor suppressor genes such as *MLH1* [29, 61, 62]. *MLH1* is a mismatch repair (MMR) gene, whose silencing is associated with the transition of SSLs to SSLDs and eventually leads to the development of CIMP-H/MSI-H CRCs [63–65]. Approximately 75% of SSLDs exhibit MSI-H, resulting from specific hypermethylation of *MLH1* [53].

Other factors associated with dysplastic changes in SSLs include the activation of the WNT signaling pathway. More than 60% of SSLDs harbor truncating mutations in *RNF43* (50%), *APC* (9%), or *ZNRF3* (7%), whereas SSLs rarely have mutations in genes involved in the WNT signaling pathway (7%) [66]. In agreement with this finding, nuclear β-catenin accumulation and MYC overexpression are present in most SSLDs, but are rare in SSLs. Low-frequency *APC* mutations in SSLs/SSLDs are in contrast to conventional adenomas, which show *APC* mutations in more than 90% of cases as an initial event in the adenoma–carcinoma sequence. Mutations in *RNF43* are observed in 86% of MLH1-deficient SSLDs, which may indicate an intimate relationship between *MLH1* promoter hypermethylation and activated WNT signaling [66]. Consistent with these results, a recent transcriptomic approach at the single-cell level in serrated polyps demonstrated that SSLs did not exhibit WNT pathway activation or a stem cell signature [67]. In contrast, SSLDs show upregulated signatures of cell proliferation and activation of MYC signaling [68]. Zhou et al. identified distinct epithelial subpopulations (Epi-1 and Epi-2) predominantly in serrated lesions [68]. The Epi-1 subset was mainly observed in SSLs and SSLDs, whereas Epi-2 was exclusively observed in TSAs (Fig. 3b). Compared with Epi-2, which was characterized by upregulated pathways of lysosome secretion and angiogenesis, the Epi-1 subset demonstrated higher expression of proliferation markers (*MKI67* and *SOX4*) and stem cell markers (*OLFM4*, *HES1*, and *JUN*), and several activated oncogenic signatures, such as MYC, G2M checkpoint, NOTCH signaling, E2F targets, and MAPK targets. The Epi-1 subcluster is more enriched in SSLDs than in SSLs [68]. These findings would explain, at least in part, the higher malignant potential of SSLs/SSLDs compared with that of TSAs, and the rapid progression of SSLDs to serrated CRCs.

Most *BRAF*-mutant SSLDs with *MLH1* hypermethylation progressed to CIMP-H/MSI-H CRCs, whereas SSLDs without loss of *MLH1* were proposed to progress to *BRAF*-mutant/MSS CRCs (Fig. 3c). *TP53* mutations are more common in *BRAF*-mutant/MSS CRCs than in *BRAF*-mutant/MSI-H CRCs [69]. MSI-H CRCs are associated with an immune-active TME, have a relatively good prognosis, and are sensitive to immune checkpoint blockage (ICB) therapy. *BRAF*-mutant/MSS CRCs tend to be poorly differentiated and mucinous, and are associated with signet ring cell morphology. MSS CRCs of serrated origin are mesenchymal and exhibit treatment-resistant and metastatic behavior with poor prognosis [70]. Importantly, MSS CRCs derived from SSLs do not necessarily require mutations in MAPK pathway genes for their development [71]. Recent data from a serrated mouse model demonstrated that intestinal epithelium-specific knockout of only two atypical protein kinase Cs (aPKCs), PKCl λ/ι and PKC, resulted in the spontaneous development of HPs, SSLs, and SSLDs in the mouse intestines [72]. These sessile serrated lesions rapidly progress to highly invasive adenocarcinomas, associated with mesenchymal activation, immunosuppression and MSS, and show poor differentiation and signet ring cell histology, resembling human BRAF-mutant/MSS CRCs [72].

Similar to CIMP-H/MSI-H CRCs, which have highly immunogenic characteristics, recent evidence has demonstrated high infiltration of cytotoxic immune cells within SSLs [67, 68]. In contrast to the infiltrating CD8^+^ T cells in SSLs, which have the potential for strong cytotoxicity, CD8^+^ T cells in SSLDs manifested overexpressed immune checkpoint genes (*PDCD1*, *CTLA4*, *TIGIT*, and *LAG3*). The expansion of M2-like anti-inflammatory macrophages was observed in serrated polyps, particularly in SSLDs and TSA. Stromal components also play a role in serrated tumorigenesis and immunosuppressive phenotypes [71, 73]. TGF-β produced by a hyperactivated tumor stroma has been proposed to skew the *BRAF*-mutant serrated precursors from the high-immune-infiltration subtype to the mesenchymal subtype with poor prognosis [74, 75]. Single-cell analyses of serrated lesions have shown that PDGFRA^+^ fibroblasts are enriched in the TME of a spectrum of serrated tumors, most evident in SSLDs [68]. PDGFRA^+^ fibroblasts secrete MMP11 to promote HBEGF cleavage and the development of serrated lesions, and display high levels of periostin, which was shown to induce immunosuppressive premetastatic niche formation. Furthermore, a recent report applying a mouse serrated CRC model driven by aPKC deficiency demonstrated that treatment with PEGylated hyaluronidase reprogrammed PDGFRA^+^ fibroblasts into an inflammatory phenotype, impaired immunosuppression, and reduced tumorigenesis and metastasis [76]. Thus, inhibition of stroma activation by use of TGF-β inhibitor or reprogramming of PDGFRA^+^ fibroblasts potentially disrupts the immunosuppressive TME of mesenchymal-serrated CRCs and induces their vulnerabilities to otherwise ineffective ICB therapy [72, 76]. In regard of a signaling pathway in fibroblast, a recent report showed that selective loss of BMPR1A resulted in upregulation of CXCL12 in fibroblasts that lead to severe histological changes in the intestines with a significant increase in stromal cell content and epithelial cell hyperproliferation, which caused formation of numerous serrated polyps [77].

## Endoscopic features for differentiating SSLs and SSLDs

### Diagnosis using white light imaging

Most SSLs are flat lesions with similar color to their surroundings and have mucus adhesion, indistinct borders, and a cloud-like surface [16, 37, 78]. However, these morphological characteristics of SSLs are not necessarily specific to SSLs but are often observed in HPs as well [79]. Endoscopic findings, such as large or small nodules on the surface and partial protrusion of SSLs, are useful indicators of dysplasia within SSLs, with an accuracy of 93.9% in the analysis of 326 SSLs [56]. Pedunculated morphology, double elevation, and central depression are associated with the presence of dysplasia in SSLs (the stage of SSLDs) [52]. As dysplasia within SSLs often exhibits a similar appearance to conventional adenomas, endoscopists need to be careful not to recognize and resect only the dysplastic area with regarding it as a conventional adenoma. Sano et al. reported a low sensitivity (46.2%) for the detection of SSLDs by morphology [56]. Furthermore, only 17.0% of the dysplastic or malignant components show a protuberant growth pattern [15]; thus, it should be noted that the dysplastic components of SSLDs sometimes do not present a characteristic morphology. The frequency of dysplasia occurrence within SSLs significantly increases with lesion size (≤ 5 mm, 0%; 6–9 mm, 6.0%; ≥ 10 mm, 13.6%) [37]. However, Bettington et al. showed that SSLDs were predominantly small polyps (54.3% < 10 mm) [15]. Murakami et al. also reported that 48 (42.9%) of 112 SSLDs or SSLs with carcinoma were ≤ 10 mm in size [16]. Such characteristics make SSLDs easy to miss and their detection requires careful colonoscopy.

### Diagnosis using narrow band imaging or chromoendoscopy

**Fig. 4:**
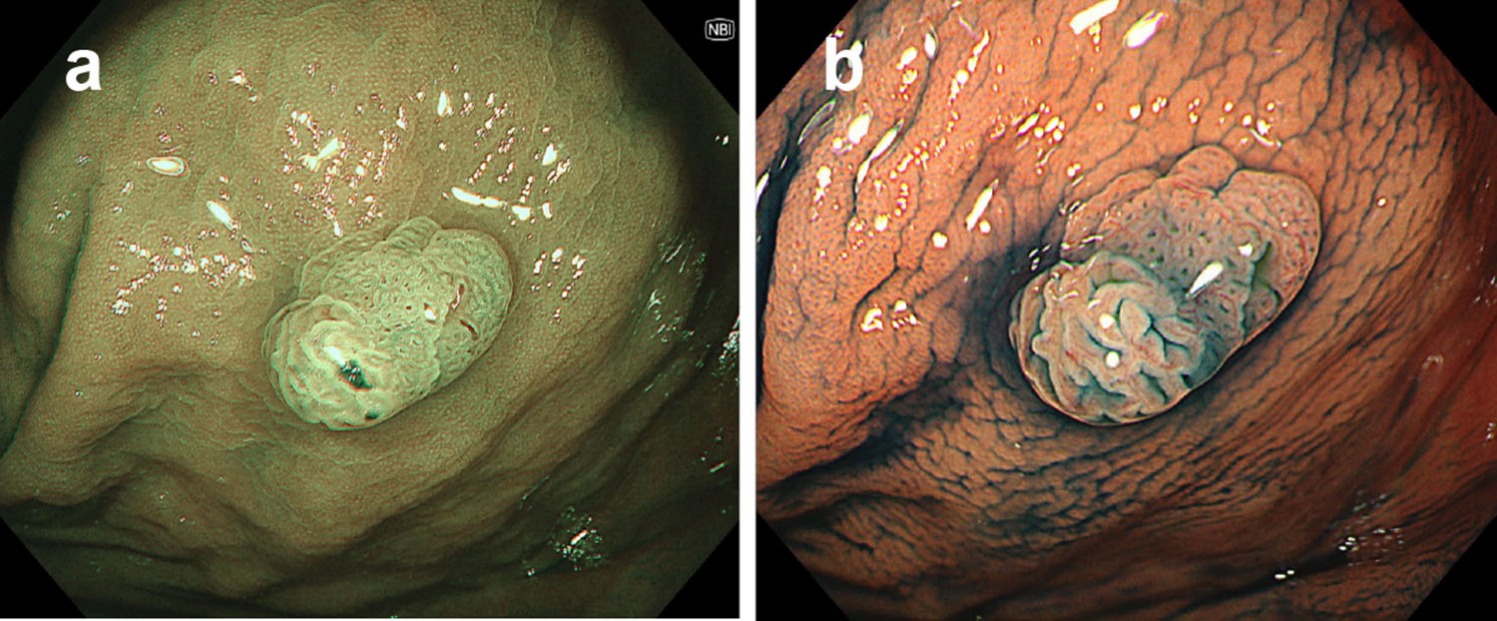
Endoscopic image of sessile serrated lesion (SSL; right of the polyp) with dysplasia (left area). **a** By magnifying narrow band imaging, small dark dots inside the openings to the crypts and varicose microvascular vessels, which are thicker than meshed capillary vessels and meandering, were found in SSL; JNET Type 2 was found in dysplasia. **b** Magnifying chromoendoscopy with indigo carmine showed the type II open pit pattern in SSL, and the type IV pit pattern in dysplasia

Narrow band imaging (NBI) with magnifying colonoscopy and chromoendoscopy is a useful strategy to distinguish between SSLs and HPs [80–87]. For example, NBI magnifying colonoscopy allows endoscopists to find small dark dots inside openings to crypts [80, 85] and varicose microvascular vessels running throughout the deep mucosal layer [83], which are both typical findings of SSLs. The type II open pit pattern (PIT) indicated by magnifying chromoendoscopy suggests the existence of a dilated crypt base, which is recognized in more than 60% of SSLs (sensitivity 66%, specificity 97%) [87] (Fig. 4).

Although there are still a limited number of reports (Table 3), both the Japan NBI Expert Team (JNET) classification [88, 89], a universal magnifying NBI classification of colorectal lesions, and the PIT classification [90–92] with chromoendoscopy are useful for identifying SSLDs. SSLs tend to exhibit JNET type 1 (JNET 1) and type II or II open PIT, while dysplasia, including adenoma or carcinoma, shows JNET type 2A, 2B, or 3, and type III, IV, or V PIT. The presence of dysplastic patterns (JNET type 2A/2B/3 or type III/IV/V PIT) within SSL (JNET type 1 or type II/open II PIT) often suggests the transition of SSLs to SSLDs or SSLs with carcinoma [52, 93–97]. In particular, applying the JNET classification to the diagnosis of SSLDs and SSLs with carcinoma had high sensitivity (83.9%), specificity (95.5%), and accuracy (94.5%) [93]. Murakami et al. reported that SSLDs with type III/IV/V PIT within SSLs showing type II PIT could be properly diagnosed with 99.4% accuracy in the analysis of 314 SSLs [52]. However, in the analysis of 201 large SSLs (≥ 20 mm in size), SSLDs exhibited neoplastic pit patterns with an accuracy of only 70.6% [95], suggesting difficulty in detecting small changes within large SSLs. Furthermore, it remains controversial whether subtle neoplastic changes within SSLDs, such as minimal deviation dysplasia or small foci with MLH1 loss, can be detected by colonoscopy. Further studies are needed to evaluate the efficacy of colonoscopy for the diagnosis of SSLDs and to develop alternative or combinatory approaches. Defining alterations in the immune and stromal components of serrated polyps by colonoscopy is still challenging, but could be considered as a supplemental strategy in the future.

**Table 3:** Endoscopic diagnostic performance for the differentiating between SSLs and SSLDs

Author	Year	Study design	Diagnostic method	SSL with dysplasia/carcinoma (*n*)	SSL without dysplasia (n)	Sensitivity	Specificity	Accuracy
Sano	2018	Retrospective	WLI (only morphology)	26 (dysplasia)	300	46.2%	97.3%	93.3%
Murakami	2017	Retrospective	WLI	41 (dysplasia), 7 (carcinoma)	414	91.7%	85.3%	85.9%
Tate	2018	Prospective	WLI + NBI (Non-magnifying)	36 (dysplasia, size ≥ 8 mm)	105 (size ≥ 8 mm)	93.9%	95.4%	95.0%
Murakami	2021	Retrospective	Magnifying NBI (JNET classification)	52 (dysplasia), 10 (carcinoma)	647	83.9%	95.5%	94.5%
Burgess	2016	Prospective	Pit pattern^*^	66 (dysplasia, size ≥ 20 mm)	135 (size ≥ 20 mm)	66.7%	72.6%	70.6%
Murakami	2017	Retrospective	Magnifying Chromoendoscopy (Pit pattern)	30 (dysplasia), 6 (carcinoma)	278	94.4%	100.0%	99.4%
Tanaka	2017	Prospective	Magnifying Chromoendoscopy (Pit pattern)	33 (dysplasia)	90	93.9%	87.8%	89.4%

^*^The use of dyes was not always required in the study

*SSL* sessile serrated lesion, *WLI* white light imaging, *NBI* narrow band imaging, *JNET* Japan NBI expert team

## Differences in therapeutic indications between SSLs and SSLDs

Endoscopic removal is used for the treatment of most SSLs and SSLDs. Cold snare polypectomy (CSP) has become the standard method for non-pedunculated colorectal polyps < 10 mm in size [98–101] because of the low risk of delayed post-polypectomy bleeding and perforation, short procedure times, and low costs. In addition, piecemeal CSP for large SSLs (≥ 10 mm) has been reported to be safe and effective. The residual rates of SSLs of ≥ 10 mm resected by CSP were lower than those of adenomas [102–107]. In an analysis of 474 SSLs of ≥ 10 mm resected by CSP or piecemeal CSP, only one case (0.2%) had residual serrated tissue identified by post-polypectomy biopsy, and no recurrence was observed [105]. However, several studies have reported that 3.7%–9.6% of lesions resected by CSP are SSLDs [107, 108]. CSP has a shallow resection depth; thus, this method is not suitable for carcinomas [99, 109–111]. As SSLDs tend to have incomplete resection owing to their obscure borders and rapid progression to carcinoma, en bloc removal with endoscopic mucosal resection (EMR) or endoscopic submucosal resection (ESD), which enables deeper resection, better pathological evaluation, and a lower recurrence rate compared with CSP, should be considered to treat endoscopically suspicious SSLDs [112–114]. Given the difficulty in accurately detecting neoplastic changes in SSLDs before resection, especially subtle dysplastic changes, the application of CSP for SSLs should be carefully evaluated in the future. Several groups, including the US Multisociety Task Force and European Society of Gastrointestinal Endoscopy, have proposed an appropriate surveillance interval after the resection of serrated polyps in published guidelines [115–117]. Since TSAs, large SSLs (≥ 10 mm), and SSLDs yield metachronous advanced neoplasia or CRC risks similar to conventional adenomas [118], these guidelines recommend 3-year intervals after polypectomy of these polyps. However, considering that SSLDs have high rates of incompletely excised lesions and rapid growth to invasive carcinoma, the surveillance interval after removal of SSLDs may need to be shorter.

## Conclusions

Research on the serrated neoplasia pathway is an evolving field and has revealed new findings in both clinical and basic science, since this pathway was defined as an alternative route to CRC a few decades ago [28]. However, despite their clinical importance, SSLDs have been overlooked because of their high miss rate during colonoscopy owing to the difficulty in identification, inconsistent recognition, and the relatively short period of this intermediate state. The lack of synchronization in the nomenclature of serrated polyps has also caused confusion between patients and providers. Updated classification of serrated polyps and recent advances in colonoscopy examination have improved the detection and proper diagnosis of SSLDs and have allowed the substantial study of these lesions macroscopically, histologically, and on a molecular basis. However, several issues must be addressed to fully understand the biology of SSLDs and to prevent their progression to CRCs, which is usually faster than that of adenomas. More in-depth morphological investigations of SSLDs, with a particular focus on the TME, are absolutely warranted to validate the findings from studies at the molecular level, including recent animal models and single-cell transcriptomes. In addition, it is vital to identify the molecular steps that determine the fate of the precursor lesions (whether SSLs/SSLDs progress to either good-prognosis immunogenic MSI-H CRCs or poor-prognosis mesenchymal MSS CRCs), considering the applicable therapeutic options including ICB therapy.
